# Generation and functional characterization of a conditional *Pumilio2* null allele

**DOI:** 10.7555/JBR.32.20170117

**Published:** 2017-12-31

**Authors:** Kai-bo Lin, Shi-kun Zhang, Jie-li Chen, Ding Yang, Meng-yi Zhu, Eugene Yujun Xu

**Affiliations:** State Key Laboratory of Reproductive Medicine, Nanjing Medical University, Nanjing, Jiangsu 211166, China.; State Key Laboratory of Reproductive Medicine, Nanjing Medical University, Nanjing, Jiangsu 211166, China.; State Key Laboratory of Reproductive Medicine, Nanjing Medical University, Nanjing, Jiangsu 211166, China.; State Key Laboratory of Reproductive Medicine, Nanjing Medical University, Nanjing, Jiangsu 211166, China.; State Key Laboratory of Reproductive Medicine, Nanjing Medical University, Nanjing, Jiangsu 211166, China.; State Key Laboratory of Reproductive Medicine, Nanjing Medical University, Nanjing, Jiangsu 211166, China.

**Keywords:** RNA-binding protein, *Pumilio2*, PUF, fertility, viability

## Abstract

The highly conserved RNA binding protein PUF (Pumilio/FBF) family is present throughout eukaryotes from yeast to mammals, with critical roles in development, fertility and the nervous system. However, the function of the mammalian PUF family members remains underexplored. Our previous study reported that a gene-trap mutation of *Pum2* results in a smaller testis but does not impact fertility and viability. Although the gene-trap mutation disrupted the key functional domain of PUM protein–PUM-HD (Pumilio homology domain), but still produced a chimeric *Pum2*-β-geo protein containing part of PUM2, raising a question if such a chimeric protein may provide any residual function or contribute to the reproductive phenotype. Here, we report the generation of a conditional *PUM2* allele, when knocked out, producing no residual PUM2 and hence a complete loss-of-function allele. We also uncovered small but significant reduction of male fertility and viability in the mutants, suggesting requirement of PUM2 for male fertility and viability.

## Introduction

The *Pumilio2* (*Pum2*) gene is a mammalian member of the classical RNA binding protein family, the PUF family (Pumilio and FBF). The PUF family genes are widely present in eukaryotes, from yeast to mammals. The *Pumilio* gene was first identified in Drosophila by Lehmann and NÜsslein-Volhard in 1987^[1]^. In *C. elegans*, FBF (for fem-3 binding factor) was reported as a homolog of Drosophila *Pumilio* in 1997^[2]^. FBF and Pumilio homologs hence constitute a large and evolutionarily conserved RNA binding protein family, thereafter called the PUF family. The PUF family is characterized by its highly conserved RNA binding domain PUM-HD (Pum Homolog Domain). PUM-HD consists of eight Puf repeats with about 40 amino acids each repeat and is essential for RNA binding by the PUF protein^[2^–^4]^. Furthermore, PUM-HD is reported to interact with other proteins^[5^–^6]^. Members of the PUF family modulate target gene expression at post-transcriptional levels *via* binding to the PBE (PUM Binding Element) motif (UGUAHAUA) in the 3′UTR of target transcripts, and thus regulate developmental processes among various organisms^[7^–^8]^.

Invertebrate members of the PUF family play significant roles in embryogenesis and germ cell development. *Drosophila* Pumilio protein controls anterior-posterior axis formation, maintenance of germline stem cells and neuron development *via *post-transcriptional regulation^[9^–^14]^. In *C. elegans*, FBF1 and FBF2 are involved in the regulation of sperm-oocyte switch, maintenance of germline stem cell and the size of germline mitotic region^[2^,^5^,^15^–^16]^. These findings suggest that highly conserved mammalian PUM proteins may also play important roles in development^[8^,^17]^.

Previously mouse *Pum2*^*XE772/XE772*^ mutation was reported to affect nervous system function and testis size but not fertility or viability in mice^[18^–^19]^. This *Pum2* mutation resulted from a gene trap mutation where the β-geo gene was inserted between exon10 and exon11 of *Pum2*. The insertion mutation led to a PUM2-β-geo fusion protein, removing most part of PUM2 protein after exon 10 including the most conserved PUM-HD domain^[18]^. While this *Pum2* allele should disrupt the key function of PUM2 protein in its ability to bind its target sequences, it remains unknown if all the other functions of PUM2 were also disrupted and if the chimeric PUM2-β-geo fusion protein might impact the phenotype of *Pum2* allele. Furthermore, recent studies reported that PUM2 together with PUM1 regulates neurogenesis^[20]^, indicating a need to generate a conditional *Pum2* allele for simultaneous study of both *Pum1* and *Pum2* in specific tissues of interest. We hence seek to generate a new loss-of-function *Pum2* allele, which removes all parts of PUM2 protein and could be used for conditionally removing *Pum2* in any tissues for future characterization of PUM family protein function.

We succeeded in establishing a complete loss-of-function allele of *Pum2* and the usability for conditional knockout of *Pum2* in the presence of Cre recombinase. Whole body knockout of *Pum2* led to reduced bodyweight and reduced testis size, similar to the previous *Pum2* gene trap allele. However, this new mutation also reduced male fertility and viability slightly, suggesting key roles of PUM2 in maintaining normal male fertility and viability.

## Materials and methods

### Animals

Mice were housed and maintained on a 12 h-light-12 h-dark cycle with free access to water and food under the specific pathogen-free conditions in Animal Core Facility of Nanjing Medical University, China. All animal experiments were approved by the Institutional Animal Care and Use Committee (IACUC) of Nanjing Medical University and Northwestern University. *Pum2* mutant mice used for analysis were on C57BL/6 (Jackson Laboratory, #000664) background.

### Generation of ***Pum2*** mutant mice

**Fig.1:**
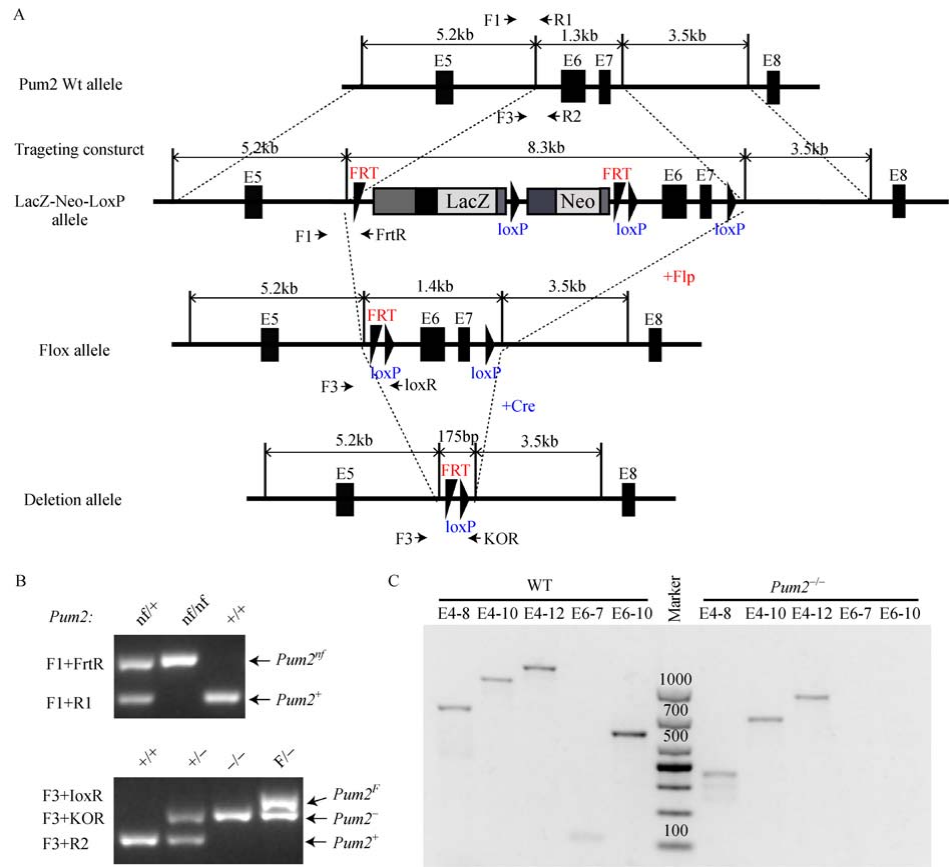
Generation of ***Pum2*** knockout mice.

The *Pum2* strain was created from ES cell clones Pum2 (EPD0270) B04, Pum2 F03, obtained from KOMP repository and generated by Welcome Trust Sanger Institute^[21]^. The cassette is composed of an FRT site followed by lacZ sequence and a loxP site. This first loxP site is followed by neomycin under the control of the human beta-actin promoter, SV40 polyA, a second FRT site and a second loxP site. A third loxP site is inserted at the position of 207 bp downstream of the targeted exons 6 and 7. The target region, exon6 and exon7, was flanked by two loxP sites and the selection cassette was flanked by *FRT* sites (***Fig. 1A***). Blastocyst injection and derivation of chimera were done at Northwestern University Transgenic Core facility. We first injected the ES cell clone into C57BL/6 blastocyst to get a chimeric mouse and backcrossed the chimeric mice to establish a colony of mice that transmitted the targeting construct *Pum2*^*nf*^ gene in the germ line. Then, the *Pum2*^*nf/+*^ mice were mated with CAG-flip (C57BL/6) (to delete the selection cassette) and Ella-Cre (C57BL/6) (to delete exons 6 and 7) to generate mouse with the *Pum2*^*F*^ and *Pum2*^–^ alleles, respectively. All offspring were genotyped by PCR using primers shown in ***Fig. 1A***.

### Genotyping of ***Pum2*** mutant mice by genomic PCR

Genomic DNA was extracted from tail tip for genotyping. Pum2-F1, Pum2-R1 and Pum2-FrtR were used for detecting the wild-type allele (*Pum2*^*+*^, 175bp) and the Frt-Neo-Frt-loxP cassette-containing allele (*Pum2*^*nf*^, 310bp). Genotyping primers for the wild-type allele (*Pum2*^*+*^, 132bp), the loxP-Exon6-Exon7-LoxP (*Pum2LoxP* hereafter referred to as *Pum2*^*F*^, 271bp) and the deletion allele (*Pum2*^–^, 210bp) are Pum2-F3, Pum2-R2, Pum2-KOR and Pum2-loxR. Target regions were amplified by GoTaq Green master mix (Promega). All primers for genotyping are presented in ***Table 1***.

**Tab.1:** PCR primers for genotyping

Primers	Sequences (5′→3′)
Pum2-F1	GCTACTCCCTTTCTTGCTTG
Pum2-R1	CCGTGAGTGAAAGAAATCTAAAC
Pum2-FrtR	CCCTTCCTCCTACATAGTTGGC
Pum2-F3	GCACAGAAAAAAACCTTTGAAAGTC
Pum2-R2	ATGGCAAAGCTCAAAATTCCACTT
Pum2-KOR	TTTGAACTGATGGCGAGCTC
Pum2-loxR	AAAACAACTATTAAATAACACCGCCTACTG

RNA extraction and reverse transcription-PCR (RT-PCR)

RNA was extracted by using TRIzol Reagent (Invitrogen) according to the manufacturer’s instructions and measured by Nanodrop 2000 (Thermo). Reverse transcription (RT) was carried out following standard procedures using random primers and PrimeScript RT Master Mix (Takara) (***Table 2***).

**Tab.2:** RT-PCR primers for ***Pum2*** mRNA

Primers	Sequences (5′→3′)
exon4-F	AATGCAATTCTTTCTCCACG
exon6-F	GTAATCAGGTACCCATGGAT
exon7-R	TTAACTGCTGAACTGTTAGTG
exon8-R	GCAGCACTAATAATATATGGAT
exon10-R	AGCAAGACTCTGACCAAAAG
exon12-R	CATTTGTGCTGCCTGTAAGA

Protein extraction and Western blot.

Testes were collected from adult *Pum2^+/+^*, *Pum2*^–*/*–^, *Pum2*^*nf/nf*^, and *Pum2*^*XE772/XE772*^ mice. All samples were lysed in RIPA buffer containing protease inhibitor (5 mg/mL final concentration). After incubation for 15 min on ice, the tissue lysates were centrifuged at 20,000 *g* for 30 min at 4°C and then the supernatant was carefully collected. The lysates were subjected to SDS-PAGE followed by immunoblotting. Proteins were separated by 12% SDS-PAGE gels and transferred to PVDF membranes (*Bio-Rad*). The membrane was blocked with 10% (w/v) skim milk powder in TBS (100 mmol/L Tris-HCl, pH7.3) containing 0.1% (v/v) Tween 20 (TBST) for 1 h and then incubated with anti-PUM2 antibody (1:500, Bethyl laboratory) or anti-β-actin antibody (1:2,000, Sigma) at 4°C overnight. After three washes (5 min each) with 1 × TBST, the membranes were incubated with a secondary antibody conjugated with horseradish peroxidase for 1 h at room temperature, followed by washing. Protein bands were detected by using ECL Western blotting reagents (Thermo) for exposure.

### Bodyweight and fertility measurement

Bodyweight was measured weekly from newborn to adult stage for the three genotypes (*Pum2*^*+/+*^, *Pum2*^*+/*–^, *Pum2*^–*/*–^). Eight-week-old males or six-week-old females were housed singly with wildtype ICR females or ICR males of proven fertility for at least 6 months. A total of 6 *Pum2*^–*/*–^, 4 *Pum2*^*+/*–^ and 3 *Pum2*^*+/+*^ mice were tested in both males and females. The litter sizes were recorded continuously.

### Histology analyses

Tissues were fixed for 24 h in Hartman’s fixative (Sigma). Paraffin embedded sections (5 μm) were stained with hematoxylin and eosin (H&E) and images were obtained with microscope (Zeiss, Axioscope) and processed using the AxioVision LE software.

### Sperm count and sperm motility assay

The number of sperm was counted *via *a hemocytometer as follows: one caudal epididymis was collected and dissected into numerous smaller pieces in 1 mL pre-warmed (37°C) 1 × PBS and followed by incubation at 37°C for 30 min to allow sperm release. Sperm motility was analyzed by using the computer-assisted sperm analysis (CASA, HamiltonThorne, TOX IVOS) system after 5 min incubation at 37°C.

## Results

### Generation of a conditional ***Pum2*** allele

*Pum2* ES clone containing the target *Pum2* allele was injected in C57BL/6 blastocyst, and the resulting chimera was crossed with C57BL/6 mice to generate *Pum2*^*nf*^ allele (LacZ-Neo-LoxP allele), a knockout allele containing neomycin cassette and loxP insertions in the *Pum2* locus (***Fig. 1A***). *Pum2*^*nf/+*^ mice were crossed to CAG-flip mice to generate *Pum2*^*F*^ allele (Flox allele) at the same time. *Pum2*^*+/*–^ mouse was generated after crossing Ella-CRE with *Pum2*^*F/F*^ mouse, confirming the usability of floxed *Pum2* allele for conditional knockout *via *Cre recombinase (***Fig. 1A***). *Pum2*^–*/*–^, *Pum2*^nf/nf^, *Pum2*^f/f^ and their heterozygotes could be distinguished *via *PCR genotyping (***Fig. 1B***).

To confirm the deletion of exon6 and exon7 in *Pum2* homozygotes, we extracted RNA and protein from adult *Pum2*^*+/+*^ and *Pum2*^–*/*–^ testes. No transcripts containing exon 6 or 7 were present in the mutant tissues as RT-PCR using primers from exon 6 or 7 failed to detect any product (***Fig. 1C***). Despite deletion of exons 6 and 7, *Pum2*^–*/*–^ mice still produced a shorter *Pum2* transcript. However this mutant transcript is predicted to only produce a greatly truncated PUM2 (191 aa; MNHDFQALALESRGMGELLPTKKFWEPD DSTKDGQKGIFLGDDEWRETAWGTSHHSMSQP IMVQRRSGQSFHGNSEVNAILSPRSESGGLGVS MVEYVLSSSPADKLDSRFRKGTFGTRDAETDG PEKGDQKGKASPFEEDQNRDLKQDDEDSK INGRGLPNGMDADCKDFNWCILSRPCSSCICAKS IYY*) without most part of PUM2 including PUM-HD, due to a reading frame shift and a premature stop codon after exon 5. Thus, we generated a novel *Pum2* allele lacking wildtype *Pum2* transcripts.

**Fig.2:**
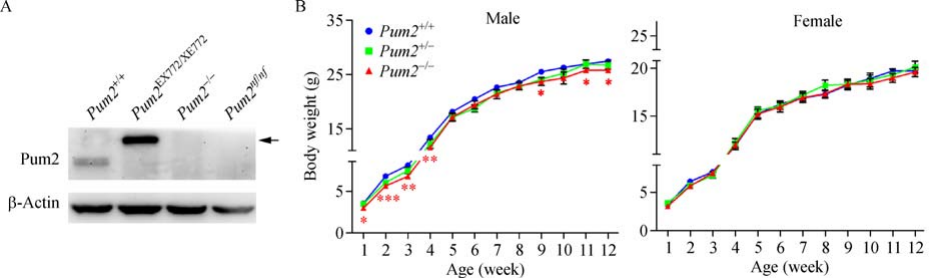
Bodyweight reduction in ***Pum2*** homozygotes.

We next determined if PUM2 was completely knocked out. We examined PUM2 expression in the testes of wildtype and three Pum2 mutants. The *Pum2*^*nf*^ allele was the initial knockout allele produced from chimeric founder mice. It is similar to the gene trap allele, disrupting the expression of *Pum2* but without signs of fusion protein. Indeed, PUM2 protein was completely absent in *Pum2*^*-/-*^ and *Pum2*^*nf/nf*^ testes whereas the PUM2-β-geo fusion protein was abundant in *Pum2*^*XE772/XE772*^ (***Fig. 2A***). Further examination of PUM2 in other tissues (brain, thymus, liver, spleen, lung, kidney and ovary) validated complete loss of PUM2 in all the tissues examined (***Supplementary Fig S1***, available online). Given our antibodies recognize only the *N*-terminus of PUM2 (100 to 150 amino acids of PUM2), failure to detect any PUM2 bands of the predicted truncated protein on Western bots indicated that no truncated or residual PUM2 protein was produced from the new *Pum2* mutant tissues. We hence generated a complete loss-of-function *Pum2* allele.

### ***Pum2*** is required for normal growth in mice

Previous study reports that *Pum2*^*XE772/XE772*^ mice show reduced bodyweight^[18]^. We found that the new *Pum2*^–*/*–^ homozygous mice were similar to wildtype in overall morphology (data not shown) and also showed reduced bodyweight from 1 week to 12 weeks of age in males (***Fig. 2B***). These data suggest that loss of *Pum2* impacted male bodyweight during postnatal mouse growth. The difference in sexes could be due to sample size and can be further examined with a larger number of animals. Indeed, for mice 20-week old or older, both male and female *Pum2*^–*/*–**^mice showed small yet significant reduction of bodyweight than their wild type counterparts, confirming bodyweight reduction was not restricted to males.

We also noticed slightly reduced number of *Pum2*^–*/*–^ (18.4%) among the pups produced from heterozygote interbreeding (***Table 3***). Although the number of offspring analyzed remained small, this significant deviation from Mendelian ratio may suggest a role of PUM2 in growth control.

**Tab.3:** ***Pum2*** genotype analysis from heterozygous intercrosses

Genotype	*Pum2*^*+/+*^	*Pum2*^*+/–*^	*Pum2*^*–/–*^
Total (266)	76	141	49*
Male (134)	40	68	26
Female (132)	36	73	23

*P 0.05

### Reduced fertility of ***Pum2***^–*/*–^ mutant males

**Fig.3:**
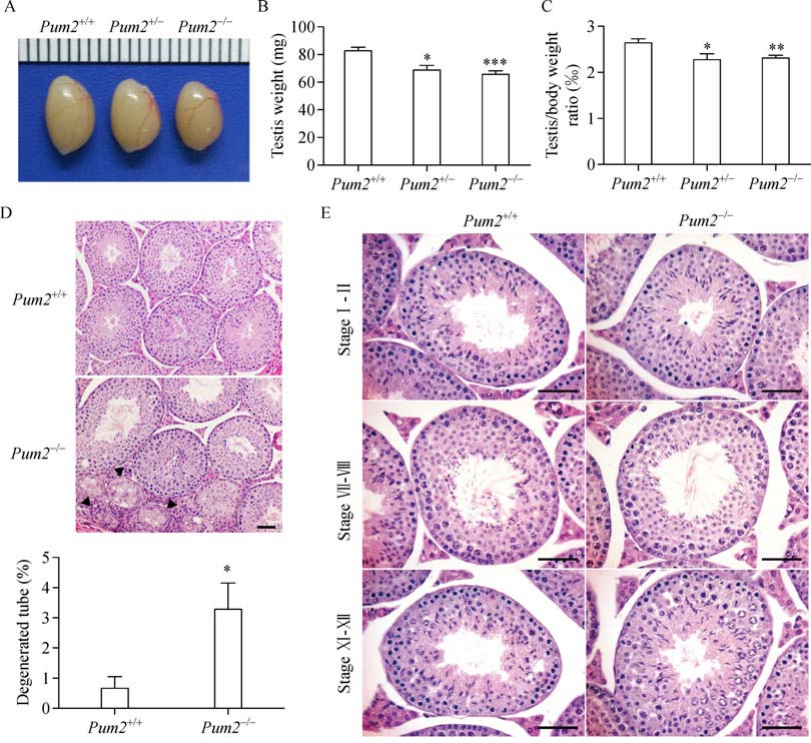
Reduced testis weight in ***Pum2***^–*/*–^ male mice.

Adult *Pum2*^–*/*–^ males exhibited smaller testes than those of *Pum2*^*+/+*^, similar to t *Pum2*^XE772/XE772^ (***Fig. 3A***). Testis weight of both *Pum2*^*+/*–^ and *Pum2*^–*/*–^ was reduced (***Fig. 3B***). To exclude the effect from the overall bodyweight reduction, we measured the ratio of testis weight over bodyweight and found that it was also significantly reduced in *Pum2*^*+/*–^ and *Pum2*^–*/*–^ mice than that of wildtype mice (***Fig. 3C***). Histological examination of mutant testes showed a small increase in the number of degenerating tubules in the testis, suggesting a possible cause of testis weight reduction (***Fig. 3D***). Examination of apoptotic cells in the testis did not reveal any significant difference between wildtype and *Pum2*^–*/*–^ (***Supplementary Fig. 2A***, ***B*** and ***C***, ******available online). Other than degenerated tubules, spermatogenesis stages were similar in both wildtype and *Pum2*^**–* / *–**^based on histological staging of the testes (***Fig. 3E***).

**Fig.4:**
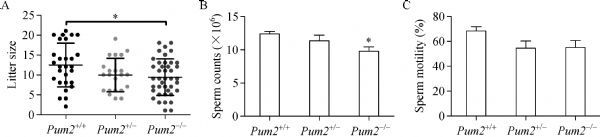
***Pum2*** null males exhibited subfertility.

We further examined the fertility of *Pum2*^–*/*–^ males, by mating 8-week-old *Pum2*^*+/+*^, *Pum2*^*+/*–^ and *Pum2*^–*/*–^ males with wildtype females. Our results showed that loss of *Pum2* also reduced male fertility (***Fig. 4A***). *Pum2*^–*/*–^ males showed reduced sperm count (***Fig. 4B***); however, sperm motility and sperm morphology *Pum2* homozygotes appeared normal (***Fig. 4C*** and ***Supplementary Fig. 2D***, available online). These data suggested that *Pum2* was required for normal male fertility.

### ***Pum2*** is dispensable for female fertility

*Pum1*, another member of Puf family, is an important translational regulator for mammalian female germ cell development^[22]^. To analyze roles of *Pum2* in female mice, 6-week-old *Pum2* mutant female mice were crossed to wildtype males. There was no significant difference in female fertility (***Supplementary Fig. 3A***, available online) and ovary weight (***Supplementary Fig. 3B***, available online). The number of developing follicles and corpus luteum in adult *Pum2*^–*/*–^ ovaries was comparable to age-matched *Pum2*^*+/+*^ ovaries (***Supplementary Fig. 3C***, available online). Further supporting this notion, superovulation experiments revealed that the number of MII oocytes retrieved from 3-month-old *Pum2*^–*/*–^ females was similar to age-matched *Pum2*^*+/+*^ females (***Supplementary Fig. 3D***, available online), suggesting that Pum2 is dispensable for female fertility, consistent with the previous report^[22]^.

Our work suggests that *Pum2*, a member of the conserved mammalian PUF family, is needed for attaining normal viability and male fertility, but is not essential for female fertility.

## Discussion

RNA binding proteins have been found to play important roles in diverse physiologic processes as well as in human diseases. Recently, highly conserved RNA binding proteins-PUMILIO proteins were reported to be important for neurogenesis, germline development, genomic stability and hematopoietic stem cell proliferation^[20^,^22^–^25]^. Physiologic function of *Pumilio* has not been fully characterized yet. Our understanding of PUM2 has mainly come from a gene trap mutation, which disrupted the critical RNA binding ability of PUM2 but still produced a PUM2-β-geo fusion protein containing N-terminal PUM2^[18]^. Thus, we constructed another *Pum2* mutant allele-*Pum2*^–*/*–^, producing no PUM2. Complete absence of wildtype *Pum2* transcripts and absence of any PUM2 residual protein indicated that we have generated a *Pum2* null allele.

We confirmed the previous reports that *Pum2* is not essential for female fertility but is required for attaining normal bodyweight and testis weight^[18^–^19^,^22]^. The small effect that *Pum2* mutation has on viability and male fertility revealed potential roles of PUM2 in growth and spermatogenesis. We failed to detect any increase in apoptosis in *Pum2* mutant testes, suggesting the reduction of testis weight may not result from apoptosis. Further investigation may be needed to pinpoint the contribution of other causes such as reduced proliferation toward reduced male fertility. PUM1 was reported to play an important role in safeguarding spermatogenesis^[26]^ and *Pum1* mutants are reduced in bodyweight^[26^–^27]^. We hence hypothesize that *Pum1* and *Pum2* may both regulate viability and male fertility together. Removal of both *Pum1* and *Pum2* altogether using conditional alleles in particular tissues such as the testis could help uncover fundamental roles of the conserved PUF family proteins.

Overall, *Pum2*^–*/*–^ mutant mice is similar to *Pum2*^*XE772/XE772*^ in that they both exhibit reduced bodyweight, testis weight and sperm count^[18^–^19]^, validating that the gene trap *Pum2*^*XE772*^ allele is a strong loss-of-function allele. However, *Pum2*^–*/*–^ males displayed male subfertility and underrepresentation of homozygotes among the progeny. Such difference could result from the allelic difference due to the presence of part of PUM2 in the chimeric protein of the gene trap mutant; alternatively, it could result from a difference in mouse strain background as *Pum2*^–*/*–**^is in C57BL/6 background, but the gene trap allele used for fertility analysis was in mixed B6 and 129 background^[18]^. Phenotypic comparison of transheterozygotes and individual homozygotes could distinguish the two possibilities. Regardless, generation of this conditional null *Pum2* allele established the functional requirement of mouse *Pum2* gene and provided a key model for future characterization of the role of the PUF family proteins in diverse biological processes.
